# Amphetamine-Induced Dopamine Release Predicts 1-Year Outcome in First-Episode Psychosis: A Naturalistic Observation

**DOI:** 10.1093/schbul/sbae111

**Published:** 2024-08-13

**Authors:** Ana Weidenauer, Ulrich Sauerzopf, Martin Bauer, Carina Bum, Cornelia Diendorfer, Irena Dajic, Lucie Bartova, Alina Kastner, Karsten Bamminger, Lukas Nics, Cecile Philippe, Marcus Hacker, Dan Rujescu, Wolfgang Wadsak, Nicole Praschak-Rieder, Matthäus Willeit

**Affiliations:** Department of Psychiatry and Psychotherapy, Division of General Psychiatry, Medical University of Vienna, Vienna, Austria; Comprehensive Center for Clinical Neurosciences and Mental Health, Medical University of Vienna, Vienna, Austria; Douglas Research Centre, Clinical and Translational Sciences Lab, Montreal, Quebec, Canada; Department of Psychiatry, McGill University, Montreal, Quebec, Canada; Department of Psychiatry and Psychotherapy, Division of General Psychiatry, Medical University of Vienna, Vienna, Austria; Comprehensive Center for Clinical Neurosciences and Mental Health, Medical University of Vienna, Vienna, Austria; Department of Psychiatry and Psychotherapy, Division of General Psychiatry, Medical University of Vienna, Vienna, Austria; Comprehensive Center for Clinical Neurosciences and Mental Health, Medical University of Vienna, Vienna, Austria; Department of Clinical Pharmacology, Medical University of Vienna, Austria; Psychosocial Services in Vienna, Vienna, Austria; Department of Psychiatry and Psychotherapy, Division of General Psychiatry, Medical University of Vienna, Vienna, Austria; Comprehensive Center for Clinical Neurosciences and Mental Health, Medical University of Vienna, Vienna, Austria; Department of Psychiatry and Psychotherapy, Division of General Psychiatry, Medical University of Vienna, Vienna, Austria; Comprehensive Center for Clinical Neurosciences and Mental Health, Medical University of Vienna, Vienna, Austria; Department of Psychiatry and Psychotherapy, Division of General Psychiatry, Medical University of Vienna, Vienna, Austria; Comprehensive Center for Clinical Neurosciences and Mental Health, Medical University of Vienna, Vienna, Austria; Department of Psychiatry and Psychotherapy, Division of General Psychiatry, Medical University of Vienna, Vienna, Austria; Comprehensive Center for Clinical Neurosciences and Mental Health, Medical University of Vienna, Vienna, Austria; Department of Psychiatry and Psychotherapy, Division of General Psychiatry, Medical University of Vienna, Vienna, Austria; Comprehensive Center for Clinical Neurosciences and Mental Health, Medical University of Vienna, Vienna, Austria; Department of Biomedical Imaging and Image Guided Therapy, Medical University of Vienna, Austria; Department of Biomedical Imaging and Image Guided Therapy, Medical University of Vienna, Austria; Department of Biomedical Imaging and Image Guided Therapy, Medical University of Vienna, Austria; Department of Biomedical Imaging and Image Guided Therapy, Medical University of Vienna, Austria; Department of Psychiatry and Psychotherapy, Division of General Psychiatry, Medical University of Vienna, Vienna, Austria; Comprehensive Center for Clinical Neurosciences and Mental Health, Medical University of Vienna, Vienna, Austria; Department of Biomedical Imaging and Image Guided Therapy, Medical University of Vienna, Austria; Center for Biomarker Research in Medicine CBmed, Graz, Austria; Department of Psychiatry and Psychotherapy, Division of General Psychiatry, Medical University of Vienna, Vienna, Austria; Comprehensive Center for Clinical Neurosciences and Mental Health, Medical University of Vienna, Vienna, Austria; Department of Psychiatry and Psychotherapy, Division of General Psychiatry, Medical University of Vienna, Vienna, Austria; Comprehensive Center for Clinical Neurosciences and Mental Health, Medical University of Vienna, Vienna, Austria

**Keywords:** dopamine release, symptoms, patients, schizophrenia, PHNO, PET, psychosis

## Abstract

**Background and Hypothesis:**

The dopamine theory of schizophrenia suggests that antipsychotics alleviate symptoms by blocking dopamine D_2/3_ receptors, yet a significant subset of patients does not respond adequately to treatment. To investigate potential predictors, we evaluated d-amphetamine-induced dopamine release and 1-year clinical outcomes in 21 antipsychotic-naive patients with first-episode schizophrenia.

**Study Design:**

Twenty-one antipsychotic-naive patients (6 female) underwent dopamine D_2/3_ receptor radioligand [^11^C]-(+)-PHNO positron emission tomography. For estimating dopamine release, scans were performed with and without d-amphetamine pretreatment. The Positive and Negative Syndrome Scale was performed at regular intervals over 1 year while receiving treatment in a naturalistic setting (Clinical Trial Registry: EUDRACT 2010-019586-29).

**Study Results:**

A group analysis revealed no significant differences in d-amphetamine-induced dopamine release between patients with or without clinically significant improvement. However, d-amphetamine-induced dopamine release in ventral striatum was significantly associated with reductions in positive symptoms (*r *= 0.54, *P *= .04; uncorrected *P*-values); release in globus pallidus correlated with a decrease in PANSS negative (*r *= 0.58, *P *= .02), general (*r *= 0.53, *P *= .04), and total symptom scores (*r* = 0.063, *P *= .01). Higher dopamine release in substantia nigra/ventral tegmental area predicted larger reductions in general symptoms (*r *= 0.51, *P *= .05). Post-amphetamine binding in putamen correlated positively with negative symptom scores at baseline (*r *= 0.66, *P *= .005) and throughout all follow-up visits.

**Conclusions:**

These exploratory results support a relationship between d-amphetamine-induced dopamine release and the severity and persistence of symptoms during the first year of psychosis.

## Introduction

Schizophrenia is a psychiatric disorder with an often severe illness course and a lifetime prevalence of approximately 1% worldwide.^1,2^ During the acute phase of the disorder—a psychotic episode—patients with schizophrenia are known to suffer from positive symptoms such as delusions and hallucinations, and from negative symptoms such as blunted affect, social withdrawal, and cognitive impairment.^3^

A large number of positron emission tomography (PET) and single photon emission computed tomography (SPECT) studies supports the dopamine theory of schizophrenia. Untreated patients with schizophrenia show increased dopamine synthesis capacity and greater amphetamine-induced dopamine release in subcortical brain areas, which is also paralleled by larger behavioral responses.^4–10^ This hyperreactivity of the dopamine system has been defined as a “state of natural sensitization”^11–13^ and has been confirmed by several PET studies.^4–10^

While dopamine synthesis capacity can be estimated by determining the uptake of the radiolabeled dopamine precursor ligand 6-[^18^F]fluoro-l-dihydroxy-phenylalanine ([^18^F] DOPA), dopamine release is approximated by radioligands acting as antagonists on dopamine D_2/3_ receptors such as [^11^C]raclopride, [^123^I]iodobenzamide ([^123^I]IBZM), or agonists like [^11^C]N-propyl-norapomorphine ([^11^C]NPA) or [¹¹C]-(+)-propyl-hexahydro-naphtho-oxazin ([^11^C]-(+)-PHNO). [^11^C]-(+)-PHNO binds selectively and with high affinity to dopamine D_2_ and D_3_ (D_2/3_) receptors and is very sensitive toward changes in extracellular dopamine.^14^

As current antipsychotic treatments act primarily at D_2/3_ receptors, their antipsychotic effects are associated with the degree of D_2/3_ receptor blockade.^15,16^ Nevertheless, approximately one-third of patients with schizophrenia does not respond adequately to antipsychotic treatment.^17^ Before neuroimaging methods became available to researchers, psychiatric disorders were mostly studied from a phenomenological rather than a biological perspective. Consequently, current prognostic markers rely on specific symptoms and clinical variables. For example, it is known that the predominance of negative symptoms,^18–20^ lower baseline illness severity^21^ low illness insight,^22^ male sex^23^ greater cognitive impairment,^24^ and longer duration of untreated psychosis^19^ are associated with poor treatment outcomes.

It has also been hypothesized that antipsychotics are more effective in patients with greater dysregulation of the dopamine system^25^: Specifically, patients with increased striatal uptake of [^18^F]DOPA were more likely to respond to antipsychotic treatment than patients who did not show this alteration.^26^ Similarly, striatal [^18^F]DOPA uptake in patients with untreated first-episode psychosis (FEP) correlated negatively with time until relapse after antipsychotic discontinuation indicating that greater dopamine dysfunction in the untreated state is associated with a shorter time to psychotic relapse after discontinuation of D_2/3_ antagonists.^27^ While one [^18^F]DOPA study showed a normalization of dopamine synthesis capacity after antipsychotic treatment in patients with psychosis in schizophrenia,^28^ another study by Jauhar et al^29^ did not find any effects of antipsychotic treatment on [^18^F]DOPA uptake. A study by Abi-Dargham et al^30^ showed that depletion of extracellular dopamine leads to a larger increase in D_2/3_ availability in patients with schizophrenia, a result which is indicative of a preexisting subcortical hyperdopaminergia. Elevated extracellular dopamine was also predictive of good treatment response of positive symptoms, which is in line with the notion that greater excess in dopamine signaling is positively predictive for the response to D_2/3_ receptor blockade_._ To our knowledge, there are no studies investigating amphetamine-induced dopamine release in this context: D_2/3_ receptor radioligand studies were used for studying antipsychotic treatment response depending on tracer occupancy or uptake but not for measuring dopamine release and its relationship to clinical outcomes in treatment-naive patients with schizophrenia.^31^

Therefore, we measured d-amphetamine-induced dopamine release using the D_2/3_ agonist radioligand [^11^C]-(+)-PHNO in 21 patients with first-episode schizophrenia (FEP) and conducted a 12-month observational exploratory study after antipsychotic treatment initiation in a naturalistic treatment setting. We hypothesized that those patients responding to an antipsychotic treatment will show higher d-amphetamine-induced dopamine release. Furthermore, we expect significant relationships between change in symptoms at follow-up and dopaminergic parameters across different brain regions.

## Methods and Materials

This study (European Clinical Trial Registry: EUDRACT 2010-019586-29) was conducted at the Department of Psychiatry and Psychotherapy, Medical University of Vienna, Austria, in accordance with regulations of federal regulatory authorities and after getting approved by the Ethics Committee of the Medical University of Vienna (EC no. 321/2010).

The main aim of the project was to quantify d-amphetamine-induced changes in binding of the dopamine D_2/3_ receptor agonist PET radioligand [^11^C]-(+)-PHNO in treatment-naive patients with FEP, and to compare d-amphetamine-induced changes in [^11^C]-(+)-PHNO binding (for the sake of simplicity henceforth referred to as “dopamine release”) to dopamine release quantified in healthy volunteers before, and after, undergoing prospective sensitization to d-amphetamine. Part of the data was recently published and show increased d-amphetamine-induced dopamine release in treatment-naive patients with FEP, supporting the hypothesis of a naturally sensitized dopamine system in patients with schizophrenia.^10^

### Measurement of d-Amphetamine-Induced Dopamine Release in FEP

Methods for the measurement of d-amphetamine-induced dopamine release and patient recruitment have been described in detail previously.^10^ In brief, treatment-naive patients with FEP were recruited at in- and outpatient units at the Department of Psychiatry and Psychotherapy. Diagnoses of an acute psychotic episode within schizophrenia or schizophreniform disorder according to DSM V^3^ were made independently by 2 senior psychiatry specialists (M.W., N.P.R.). Patients had to be able to understand all procedures and risks of the study and gave written informed consent before participating in the study. With the exception of nicotine, caffeine, and occasional alcohol use, patients with a history of 5 or more lifetime exposures to drugs of abuse were excluded from enrollment. All participants underwent urine drug screens at inclusion and before each PET scan.

Synthesis of the dopamine D_2/3_ receptor agonist radioligand [^11^C]-(+)-PHNO was performed using a protocol developed along methods described by Wilson et al^32^ at the Department of Biomedical Imaging and Image-guided Therapy, Medical University of Vienna.^33,34^

Participants were administered a 90-min [^11^C]-(+)-PHNO PET scan in a GE Advance (General Electric Medical Systems, Milwaukee, WI) PET scanner without prior intervention and, on a separate day, another PET scan 90–120 min after oral administration of 0.4 mg/kg body weight d-amphetamine (Attentin, MEDICE Arzneimittel GmbH, Iserlohn, D). Raw data were reconstructed by filtered back projection to produce attenuation-corrected dynamic images consisting of 15 consecutive 1-min frames followed by 15 five-min frames. After frame-wise motion correction, dynamic PET images were co-registered to T1-weighted magnetic resonance images with AFNI software^35^ by applying a co-registration matrix obtained with averaged PET images. Images were inspected for quality and defective scans were excluded from the analysis.

Regions of interest (ROIs) for the caudate nucleus, putamen, ventral striatum, globus pallidus, and substantia nigra/ventral tegmental area were delineated in a semiautomated way using the software package ROMI.^36^ High uptake and favorable kinetics of [^11^C]-(+)-PHNO in these ROIs allow reliable quantification of D_2/3_ receptor binding within the duration of a PET scan.^37–39^ Cerebellum (avoiding midline structures) was used as reference region. Time activity curves were extracted from ROIs and the cerebellum and non-displaceable binding potential values (BP_ND_^40^) were calculated employing the simplified reference tissue model as implemented in PMOD V3.6 software (PMOD Technologies Ltd, Zurich, Switzerland). Dopamine release was estimated from calculated BP_ND_ values as follows:


$$\begin{array}{l} \frac{BP_{NDbaseline}-BP_{NDpost\text{-}D\text{-}amphetamine}}{BP_{NDbaseline}}\times 100 \end{array}$$

(ΔBP_ND_; for the sake of simplicity henceforth termed dopamine release).

### Follow-up Visits

After undergoing both PET measurements, the so far treatment-naive FEP patients received antipsychotic treatment according to international treatment guidelines.^41^ Personalized treatment decisions were based on symptoms, tolerability, and treatment response in a naturalistic setting. Patients were followed up for 12 months at regular intervals (1 week, 2 weeks, 4 weeks, 6 weeks, 3 months, 6 months, and 12 months). Additional antidepressant, anxiolytic, or other psychopharmacological treatments were freely prescribed according to clinical judgements of the treating specialists. At each follow-up visit, scores of the Positive and Negative Symptoms of Schizophrenia scale (PANSS^42^) were assessed and recorded. Moreover, clinical interviews were conducted to obtain information on the administration of and adherence to psychopharmacologic and other medications, concomitant and non-pharmacologic treatments, and possible exposure to drugs of abuse. Chlorpromazine equivalents were calculated for antipsychotic drugs as published previously.^43,44^ Response was defined as ≥50% reduction in PANSS total scores at month 3.

### Statistical Analysis

The software packages R (version 4.2.2) and SPSS were used for statistical analysis.^45^ To compare d-amphetamine-induced dopamine release between patients with and without clinical improvement, patients were divided into 2 groups depending on significant clinical improvement as defined by a reduction of ≥50% PANSS total score at month 3. We chose to set our focus on symptom reduction at month 3, as this is a time point where a stable antipsychotic treatment regime has usually been established and response can already be safely and reliably evaluated. There is no consistency in previous literature dealing with prediction of treatment response based on dopaminergic markers, which evaluated patients after 4–6 weeks,^21^ 6 weeks,^30^ 4 and 8 weeks,^26^ or 6 months.^29^ At last, we also chose the time point of treatment evaluation at month 3 because symptom remission at week 12 predicts long-term recovery.^46^ Differences in dopamine release between groups with and without clinical improvement were tested with 2-sided *t*-tests for all ROIs. Reduction of PANSS total scores and subdomains were correlated with dopaminergic parameters in the respective ROIs by means of Pearson-Product moment correlations for each time point separately. In order to control for sampling bias and the risk of type I errors in our largely observational dataset, we used complementary permutation tests^47^ for determining statistical significance of the correlations. Given the exploratory nature of the study, significance level was not adjusted for multiple testing. In addition to the analysis of conventional PANSS subscales, the associations between d-amphetamine-induced dopamine release and symptom change were explored employing the 5-factor solution of the PANSS proposed by Marder et al.^48^

To ensure the robustness of our results and an unbiased approach, we calculated mixed linear models to examine the association between dopaminergic parameters (dopamine release and [^11^]-(+)-PHNO BP_ND_) and symptom reduction across all time points and ROIs. Sex and age were entered as covariates. First, we performed a linear mixed model for PANSS total score and each symptom domain separately. PANSS score reductions were entered as dependent variable and dopamine release as the independent variable. Region and subject ID were entered as random factors and visit as fixed factor. The interaction effect of dopamine release and visit on PANSS reduction was of main interest. Thereafter, the same was performed for each region separately. Here, symptom severity/reduction was the dependent variable and dopamine release/[^11^]-(+)-PHNO BP_ND_, calculated for each region separately, was the independent variable with visit as fixed factor and subject as random factor. Symptom severity/reduction was the dependent variable and dopamine release/[^11^]-(+)-PHNO BP_ND_, calculated for each region separately, was the independent variable with visit as fixed factor and subject as random factor. Sex and age were entered as covariates.

## Results

### Patient Characteristics

Of the 21 included patients the majority (*n* = 14) completed all 7 follow-up visits until month 12. All patients completed follow-up visit 1. Nineteen patients remained in the study until the fifth follow-up visit (month 3) while 16 patients were examined at month 6. After 1 year at the last follow-up visit we were able to examine 14 patients. Reasons for drop-out were withdrawal of the informed consent without giving a specific reason. Three patients were lost to follow-up. Two patients moved abroad. At each time point treatment response was evaluated and was defined as reported in table 1.

**Table 1.** Demographic table. Mean ± SD

Sex		6 F, 15 M						
Age (years)		25.1 ± 6.3						
Illness duration (weeks)		47.1 ± 63.4						

PANSS scores								
	Baseline *n* = 21	w1 *n *= 21	w2 *n *= 19	w4 *n *= 19	w6 *n *= 19	m3 *n *= 19	m6 *n *= 16	m12 *n *= 14
Positive	21.3 ± 6.7	18.6 ± 6.6	16.9 ± 7.5	14.8 ± 6.9	14.3 ± 6.5	13.5 ± 6.8	14.4 ± 7.3	14.8 ± 8
Negative	20.1 ± 6.1	19 ± 5.4	17.1 ± 5.5	16.1 ± 7	16.5 ± 7.7	16.3 ± 8.1	19.6 ± 10	17.1 ± 8.8
General	40.7 ± 9.4	36 ± 9.3	32.8 ± 10.5	26.7 ± 12.5	29.5 ± 12.3	28.3 ± 13	33.6 ± 12.4	31 ± 31.6
Total	82.2 ± 16.9	73.5 ± 17.5	66.8 ± 19.7	57.2 ± 26.8	57.3 ± 27.2	55.2 ± 27.9	67.6 ± 26.5	62.9 ± 28.7
CPZeq	0	139.3 ± 129.6	245.2 ± 234.1	322.6 ± 398.4	317.9 ± 402.1	339.3 ± 413.9	244.5 ± 335.3	210.2 ± 279

*Note*: SD, standard deviation; PANSS, Positive and Negative Symptom Scale; w1, week 1; w2, week 2; w4, week 4; w6, week 6; m3, month 3; m6, month 6; m12, month 12; CPZeq, chlorpromazine equivalents of antipsychotic treatment.

As described in the original manuscript^10^ 3 patients had already received antipsychotic treatment before study inclusion, which consisted of sporadic exposure to olanzapine, aripiprazole, or quetiapine and had been stopped at least 2 months before study participation and was below the predefined threshold of 50 lifetime haloperidol equivalents.

Nineteen of the 21 included patients were hospitalized at the inpatient unit of the Department of Psychiatry and Psychotherapy of the General Hospital of Vienna, Austria, at least once before study inclusion and/or during the follow-up phase due to clinical exacerbation. After PET scanning patients received risperidone, olanzapine, aripiprazole, clozapine, and quetiapine as well as long-acting injectables of paliperidone and aripriprazole in different doses. The respective chlorpromazine equivalents for time points months 3, 6, and 12, for which main statistical analyses were performed, were calculated as published previously.^43,44^ To manage other symptoms like affective symptoms, sleep disturbances, and anxiety non-antipsychotic medication including mirtazapine, vortioxetine, sertraline, escitalopram, citalopram, bupropione, venlafaxine, fluoxetine, trazodone, zolpidem, lorazepam, clonazepam, diazepam, and pregabaline was administered.

Of the 21 patients 4 received long-acting injectable antipsychotics, 10 patients accepted an oral antipsychotic treatment, while 7 patients could not adhere to a stable treatment regimen or did not make use of any antipsychotic treatment at all due to poor illness insight and lack of willingness to accept treatment despite repeated psychoeducational and motivational measures at each follow-up visit or intermittent clinical controls in-between study visits. Seven of the 19 patients who were evaluated at month 3 fulfilled treatment response criteria as defined by a ≥50% PANSS total score reduction.

### Course of the Disorder in Patients With FEP

**Fig. 1.**
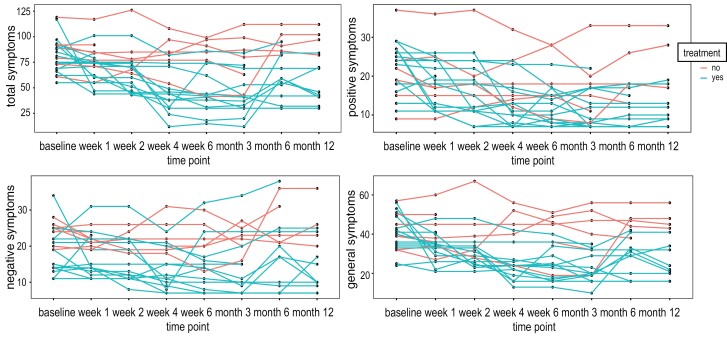
The illness course of all 21 patients with first-episode psychosis (FEP) quantified with the PANSS scale. Scores of the positive, negative, and general subdomains are shown as well as the total PANSS score. Patients without an adequate treatment are displayed (*n* = 7) while patients, who accepted an antipsychotic treatment regime (*n* = 14) are shown. The 2 patients, who were lost to follow-up are included in this plot.

PANSS scores were evaluated at each visit. In the panels of figure 1, symptom courses subdivided by PANSS subscores are shown. Patients not adhering to an adequate treatment during the follow-up visits were included in the *no treatment* group in the analysis and are marked as such in figure 1. Regarding the illness course we descriptively show how patients without adequate antipsychotic treatment display an unfavorable disease outcome in all symptom domains of the PANSS.

### d-Amphetamine-Induced Dopamine Release and Treatment Response

**Fig. 2.**
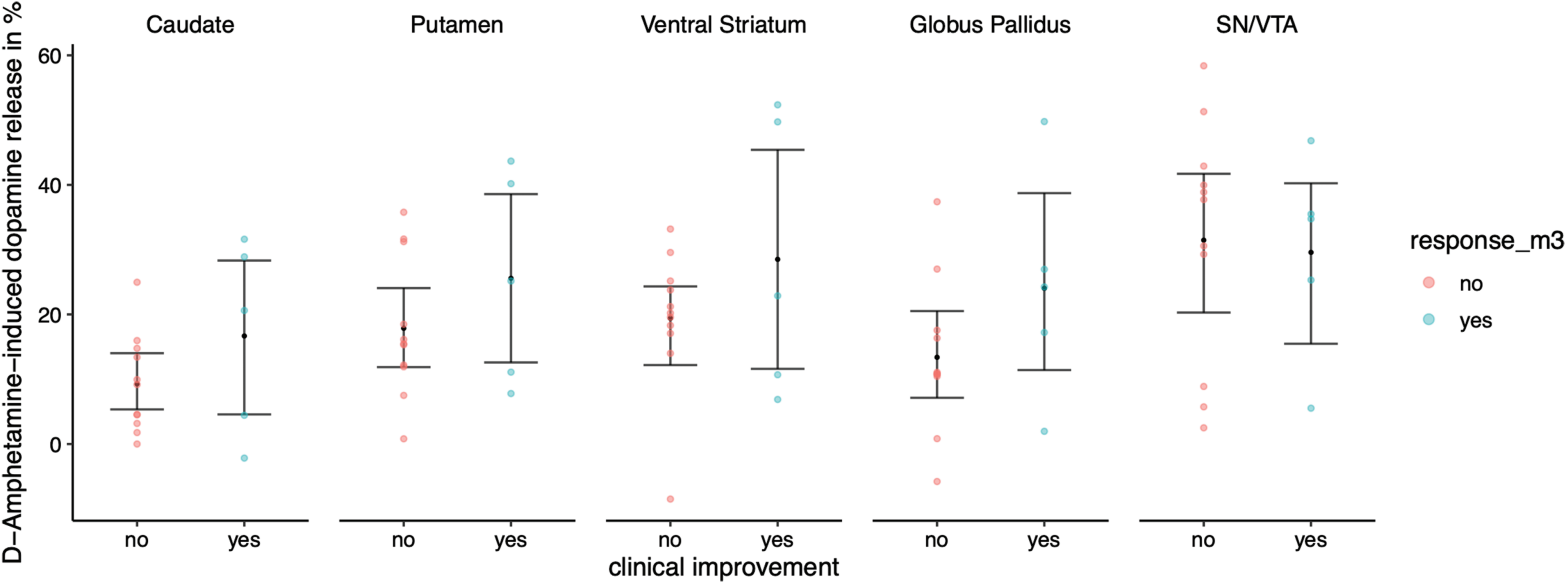
Sixteen patients (5 patients with improvement, 11 without significant improvement). From the group without improvement 5 did not accept adequate antipsychotic treatment and 6 had sufficient antipsychotic medication. Although not significant, there is a pattern of a higher d-amphetamine-induced dopamine release in patients with significant clinical improvement at month 3.

In the next step, we divided patients by significant clinical improvement (≥50% total PANSS score reduction) and those without significant clinical improvement and compared dopamine release between the 2 collectives in the respective ROIs. Of the 19 patients who attended the follow-up visit at month 3, 19 scans showed sufficient quality and were used for the analysis of treatment response and dopamine release. Of these 16 patients, 5 achieved significant clinical improvement. A pattern showing higher dopamine release in the improved individuals is depicted in figure 2, however, without statistical significance. When the analysis was performed without patients not receiving adequate treatment similar results were observed (see supplementary table 1).

### d-Amphetamine-Induced Dopamine Release and Symptom Reduction

**Fig. 3.**
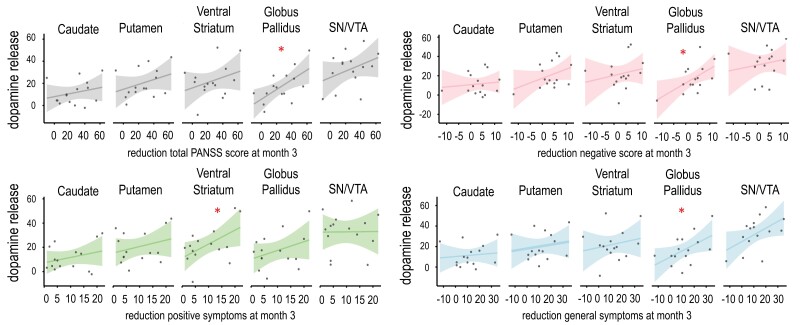
Correlations between d-amphetamine-induced dopamine release and symptom reductions of each PANSS subdomain. * indicates *P* < .05.

In the next step, we calculated correlations of baseline dopamine release and symptom improvement for the PANSS scale subdomains and total score at month 3 (figure 3). Significant correlations were found for the ventral striatum and the reduction of positive symptoms (*r* = 0.54, *P* = .04) and for the globus pallidus and negative symptom reduction (*r* = 0.58, *P* = .02). Decrease of general symptoms showed a significant positive relationship with dopamine release in the globus pallidus (*r* = 0.53, *P* = .04) and, on a trend level, the SN/VTA (*r* = 0.51, *P* = .05). Total PANSS score reduction correlated significantly with dopamine release in the globus pallidus (*r* = 0.63, *P* = .01). Permutation tests confirmed the results of these correlational analyses (ventral striatum and positive symptom reduction: *r* = 0.54, *P*_*corr*_ = .046; GP and negative symptom reduction: *r* = 0.58, *P*_*corr*_ = .03; general symptom reduction: *r* = 0.53, *P*_*corr*_ = .04; total score reduction: *r* = 0.63, *P*_*corr*_ = .006; SN/VTA and general symptom reduction: *r* = 0.51, *P*_*corr*_ = .04). The same analyses were performed for all other time points and showed a similar pattern. However, if strict correction for multiple testing were applied the results would not withstand.

The relationship between d-amphetamine-induced dopamine release and symptom change was also explored employing the 5-factor solution of the PANSS proposed by Marder et al.^48^ Here, symptom reduction in the positive symptom factor was associated with dopamine release in VST and GP (see supplementary figure 1a); and reduction in the negative symptom factor with dopamine release in GP and SN/VTA (see supplementary figure 1b). Symptom reduction in the disorganized thought factor was associated with dopamine release across all investigated brain regions (see supplementary figure 1c), while a reduction in the depressive symptom factor was associated with dopamine release in SN/VTA (see supplementary figure 1b) and reduction in the hostility/excitement factor was associated with dopamine release in GP (see supplementary figure 1e). All correlations were positive, indicating stronger dopamine release being associated with better outcomes across all 5 symptom factors.

The association between dopamine release and PANSS symptom reduction was analyzed using mixed linear models, first across all regions, and then for each brain region separately. There was a significant interaction effect of dopamine release across all brain regions and visit on PANSS total symptoms (*F*_7, 544.12_ = 6.08, *P* < .0001). The same was true for reductions in positive (*F*_7, 544.06_ = 5.95, *P* < .0001), negative (*F*_7, 544.31_ = 4.34, *P* = .0001), and general symptoms (*F*_7, 544.11_ = 4.85, *P* < .0001). The analysis of dopamine release in VST and reductions in positive symptoms across all time points showed a significant association (*F*_1, 13.52_ = 8.74, *P* = .01). Adding sex and age as covariates did not significantly change the results (sex: *F*_1, 12.61_ = 8.0, *P* = .02; main effect of sex: *F*_1, 12.93_ = 0.2, *P* = .66, age: *F*_1, 12.56_ = 8.14, *P* = .01, main effect of age: *F*_1, 12.61_ = 0.12, *P* = .73). The same was found for reductions in total PANSS scores and dopamine release in GP (*F*_1, 14.02_ = 15.88, *P* = .001). Here as well, the covariates sex and age did not significantly change the results (sex: *F*_1, 13.12_ = 12.38, *P* = .004; main effect of sex: *F*_1,13.2_ = 0.02, *P* = .99, age: *F*_1, 12.95_ = 11.19, *P* = .005, main effect of age: *F*_1,12.7_ = 0.33, *P* = .58). A significant association was also observed with respect to dopamine release in GP and reduction in negative (*F*_1,13.82_ = 5.20, *P* = .00027) and general symptoms (*F*_1, 14.00_ = 7.44, *P* = .02). Covariates sex and age shifted the association with negative symptoms to trend-level significance (sex: *F*_1,12.96_ = 4.41, *P* = .06, main effect of sex: *F*_1,13.1_ = 0.15, *P* = .7, age: *F*_1,12.65_ = 3.77, *P* = .07). The relationship with general symptoms remained significant (sex: *F*_1, 13.01_ = 6.05, *P* = .03, main effect of sex: *F*_1,13.1_ = 0.08, *P* = .78, age: *F*_1,12.96_ = 5.53, *P* = .04, main effect of age: *F*_1,12.81_ = 0.66, *P* = .43).

**Fig. 4.**
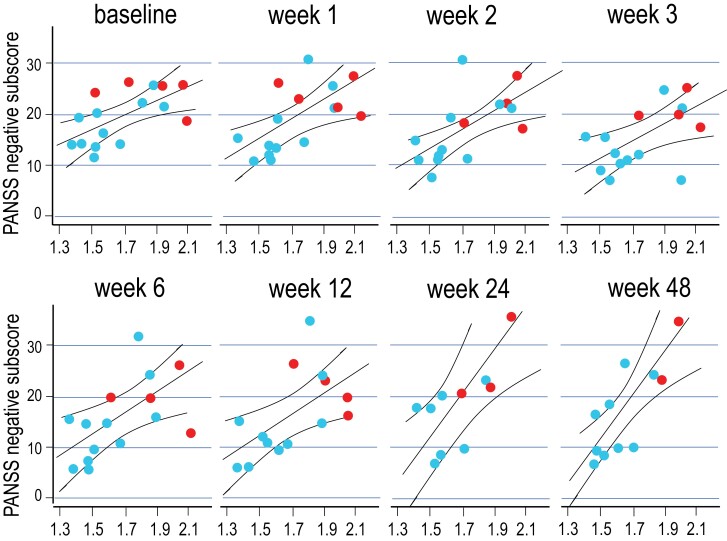
Binding of [^11^C]-(+)-PHNO to dopamine D_2/3_ receptors after d-amphetamine in the right putamen and PANSS negative symptom scores in antipsychotic-naive patients with FEP before initiation of treatment (baseline) and during a 1-year follow-up under naturalistic treatment conditions; patients with adequate adherence to antipsychotic treatment and patients with no or minimal adherence to antipsychotic treatment are shown.

As reported previously,^10^ an exploratory analysis on the relationship between psychopathological measurements and PET indices of dopamine function showed a strong correlation between the severity of negative symptoms and [^11^C]-(+)-PHNO BP_ND_ values after d-amphetamine administration in the putamen. The correlation was particularly strong in the putamen of the right hemisphere. Thus, here we tested for correlations between post-d-amphetamine [^11^C]-(+)-PHNO BP_ND_ values in the right putamen and PANSS negative subscores measured repeatedly for 1 year after the PET examination. The relationship between D_2/3_ receptor binding and expression of negative symptoms observed before treatment initiation (*r *= 0.66, *P* = .005) was found to be remarkably stable throughout all follow-up visits (minimum Pearson correlation coefficient: *r *= 0.59, *P* = .022 at month 3; maximum: *r *= 0.83, *P* = .001 1 year after PET scans; see figure 4). This is, of course, also reflecting the stability and endurance of negative symptoms^49^ that were present at first clinical manifestation of full-blown psychosis in this patient cohort (see figure 1). We also confirmed this result in a mixed linear model across all time points and found a significant relationship between post-amphetamine BP_ND_ in the putamen and negative symptoms (*F*_1,14.84_ = 7.56, *P* = .01). Including covariates in the model did not change the results (sex: *F*_1, 13.82_ = 8.38, *P* = .01; main effect of sex: *F*_1,13.83_ = 3.63, *P* = .08, age: *F*_1, 13.98_ = 9.26, *P* = .01, main effect of age: *F*_1, 13.721_ = 0.35, *P* = .56). The relationship between post-amphetamine [^11^C]-(+)-PHNO BP_ND_ values in the putamen and negative symptom severity throughout the follow-up period was also present when adopting the negative symptom factor of the 5-factor solution proposed by Marder et al (see supplementary figure 2).

## Discussion

This study in patients with FEP assessed the predictive potential of d-amphetamine-induced dopamine release before initiation of antipsychotic treatment for clinical outcome on a trajectory of 1 year. Dopamine release at baseline predicted clinical outcome and may therefore be considered a potential biomarker in the treatment of FEP. Previous PET-imaging studies have used a dopamine depletion paradigm together with the D_2/3_ receptor antagonist radioligand [^11^C]raclopride in order to estimate extracellular dopamine levels^8^ or the dopamine precursor radioligand [^18^F]FDOPA^5^ to quantify dopamine synthesis and uptake capacity, while the present study quantified the effects of d-amphetamine challenge on binding of the D_2/3_ receptor agonist radioligand [^11^C]-(+)-PHNO. The 3 methods are complementary and their specific outcome parameters reflect different aspects of subcortical dopamine functioning. Nevertheless, the results obtained with all 3 methods are consistent with the notion that increased dopamine signaling predicts better treatment response and clinical outcome in patients with schizophrenia.

Our work included one of the largest samples of mostly treatment-naive patients with FEP with a follow-up phase extending over 1-year after measuring d-amphetamine-induced dopamine release by means of [^11^C]-(+)-PHNO PET before initiating individualized guideline-oriented antipsychotic treatment within a naturalistic study design. Despite patients with FEP being a hard-to-study sample, two-thirds (*n* = 14) attended all study visits, even those not adhering to an adequate antipsychotic treatment regimen. Our sample reflects the usual treatment adherence and response rates of patients with schizophrenia,^50^ as poor illness insight and treatment adherence are hallmarks of schizophrenia. Therefore, as expected, not accepting or not adequately adhering to treatment was associated with higher PANSS scores over the course of the follow-up period.

Our main emphasis was set on treatment response at month 3 due to the fact that symptom remission at week 12 predicts long-term recovery.^46^ We nevertheless examined other time points and confirmed a consistent pattern, as we observed positive correlations in ROIs between symptom reduction and dopamine release. This corroborates dopamine release as a stable treatment response biomarker. ROIs for which the most significant results were found were the ventral striatum, the putamen, and the globus pallidus. Remarkably, the latter region was characterized by the largest difference in BP_ND_ values compared to healthy subjects in our original publication, supporting the concept of natural sensitization of the dopamine system in patients with FEP.^10^ The results of the analysis adopting the 5-factor model of PANSS symptoms^48^ were in good agreement with those obtained in canonical PANSS positive and negative subscores. This supports the internal validity of our measurements. In addition, the analysis supports an important role of dopamine in the pathogenesis of cognitive/disorganized symptoms in schizophrenia.

Considering the positive correlation between d-amphetamine-induced dopamine release and clinical improvement in our cohort, and the lack of correlation between hyperdopaminergia and positive symptoms in the untreated state, it can be speculated that other neurochemical pathologies dilute the association of positive symptoms and hyperdopaminergia across the whole sample, but that those subjects who displayed subcortical hyperdopaminergia will respond well to antipsychotic treatment. Our findings support the theory that a conventional, mostly D_2/3_ receptor targeting treatment, is effective only in patients with schizophrenia with a dopamine-based pathology.^29^

The link between dopaminergic dysfunction and treatment response was further confirmed by authors, who compared clozapine responders with clozapine-resistant patients and found higher dopamine synthesis capacity only in striatal regions of responders.^51^ Psychostimulant drugs, most noteworthy d-amphetamine, have been investigated as “pharmacological probes” in the diagnosis of endogenous schizophrenic psychosis. Patients suffering from schizophrenia frequently display exaggerated behavioral effects after ingestion of such compounds.^4^ Roughly 40% of the patients covered by the review of Lieberman et al^4^ displayed a psychotogenic response to a psychostimulant challenge. These numbers lie within the general range of prompt response to antipsychotic treatment in schizophrenia, with one-third to two-thirds of patients displaying treatment resistance.^52,53^ Treatment-resistant schizophrenia has also been conceptualized as non-dopaminergic schizophrenia.^54^ In line with the literature, in our data, roughly a third of the patient population displayed insufficient response to antipsychotic treatment despite adequate adherence. In light of our findings, the concepts of psychostimulant provocation may yet prove a useful tool in the selection of the appropriate pharmacological treatment, in particular when combined with neurochemical measures less prone to subjective rating errors than clinical observation alone.

As shown in figure 1, patients with the most intense negative symptoms were also those who were classified in retrospect as having received no adequate treatment with antipsychotics during the follow-up period (*n* = 5). Thus, it is conceivable that the persistent relationship between [^11^C]-(+)-PHNO BP_ND_ values in the putamen and negative symptom expression may arise from a lack of clinical improvement secondary to antipsychotic nonadherence. However, when restricting the analysis to the patient subsample who *did receive* adequate antipsychotic treatment, results remained statistically significant and essentially unchanged. In accordance with our findings, a recent PET study observed negative correlations between dopamine synthesis capacity and severity of negative symptoms in 2 independent cohorts of patients with schizophrenia.^55^ Moreover, a study in individuals at clinical high risk for psychosis has similarly observed a relationship between negative symptoms and lower availability of extracellular dopamine.^56^ Despite the fact that the 2 PET-imaging methods reflect 2 distinct components of dopamine signaling (binding of [^11^C]-(+)-PHNO to postsynaptic dopamine D_2/3_ receptors is primarily determined by levels of extracellular dopamine,^57^ while [^18^F]FDOPA uptake primarily reflects presynaptic dopamine synthesis and storage capacity), and despite the fact that Weidenauer et al and Eisenberg et al studied different cohorts of patients with schizophrenia (drug-naive patients with FEP and drug-free patients with a mean duration of illness of 7 ± 5 years), both studies indicate, with remarkable anatomical overlap, that negative symptoms are linked to a deficiency in dopamine signaling in the putamen.

Further studies are surely needed in order to confirm that a dopamine deficiency in circumscribed subcortical pathways has a pathogenic role in the expression of negative symptoms of schizophrenia. However, to accept, even in a preliminary way, the concept that excess and deficiencies in dopamine signaling may exist side by side, could help to change our current view on nonadherence to treatment, a problem that clinicians face particularly often in the treatment of patients with schizophrenia. Today, it is well established that occupancy of 60%–80% of subcortical D_2/3_ receptors by antipsychotics is needed for achieving satisfactory therapeutic response. But if, as the aforementioned PET studies suggest, negative symptom expression depends linearly on dopamine availability in selected parts of the dopamine system, antipsychotics, by further reducing signal transduction in these areas, will make negative symptoms even worse.

### Limitations

The sample size of 21 individuals is small, however taking into account the strenuous imaging procedures and the difficult-to-investigate population of medication-naive patients with FEP, it falls within typical ranges of PET-imaging studies. Nevertheless, we may have had insufficient power to detect phenomena of small effect size. With regards to scanning time, a previous study in nonhuman primates by Girgis et al suggests that using the simplified reference tissue model the selectivity of clozapine for D_3_ receptors could not be reliably detected when applied to 90 min PET data, and that a longer scanning time of 110 min may be a more sensitive approach.^58^ However, the first human experiments of measuring d-amphetamine-induced dopamine release^14,39^ and our own test-retest sample^10^ showed reliable measurements with 90 min PET scans even in D_3_ rich regions, and, as Girgis et al point out, it remains to be seen if a similar relationship is observable in data acquired in human subjects.^58^

Furthermore, as the naturalistic design mirrors clinical reality it inherits the limitation that patients received different types and doses of antipsychotics and were also prescribed other psychopharmacological treatments to manage co-occurring psychiatric symptoms. This has to be mentioned as a limitation of this study as it is hard to eliminate possible confounding effects of these circumstances. Furthermore, due to the exploratory nature of the study we did not perform corrections for multiple testing. However, there are stable patterns in our data across time points and brain regions and our results were confirmed by calculating mixed linear models. Blinding is a crucial issue for prospectively studying clinical interventions. However, our study is concerned with the mechanisms of pathogenesis and treatment of psychosis. The methodology using no intervention or amphetamine instead of placebo or amphetamine was chosen deliberately: Using placebo induces a significant release of dopamine, which we have shown in early studies using the highly sensitive radioligand [^11^C]-(+)-PHNO.^14^ This inherently reduces the size of the target effect, amphetamine-induced dopamine release. Thus, we do not deem placebo a suitable control condition. Regarding antipsychotic treatment, it would not have been feasible to administer only placebo to acutely ill FEP patients for a whole year.

## Supplementary Material

Supplementary material is available at https://academic.oup.com/schizophreniabulletin/.

sbae111_suppl_Supplementary_Material
