# Correction to: Amphetamine-Induced Dopamine Release Predicts 1-Year Outcome in First-Episode Psychosis: A Naturalistic Observation

**DOI:** 10.1093/schbul/sbae200

**Published:** 2024-11-30

This is a correction to: Ana Weidenauer, Ulrich Sauerzopf, Martin Bauer, Carina Bum, Cornelia Diendorfer, Irena Dajic, Lucie Bartova, Alina Kastner, Karsten Bamminger, Lukas Nics, Cecile Philippe, Marcus Hacker, Dan Rujescu, Wolfgang Wadsak, Nicole Praschak-Rieder, Matthäus Willeit, Amphetamine-Induced Dopamine Release Predicts 1-Year Outcome in First-Episode Psychosis: A Naturalistic Observation, *Schizophrenia Bulletin*, 2024;, sbae111, https://doi.org/10.1093/schbul/sbae111

In the version originally published the grant KLI958-B was omitted. The article has been updated online to include this grant.

