# Supplementary material for: Amphetamine-Induced Dopamine Release Predicts 1-Year Outcome in First-Episode Psychosis: A Naturalistic Observation
Source: Schizophr Bull. 2024 Aug 13;51(1):159–69. doi: 10.1093/schbul/sbae111 (PMC11661945; doi:10.1093/schbul/sbae111)
Supplement: sbae111_suppl_Supplementary_Material [file sbae111_suppl_supplementary_material.docx]

**Supplementary Material**

| **Supplementary Table 1.** Percent reductions in regional [^11^C]-(+)-PHNO BPND values at baseline of 16 patients with FEP, for whom scans of reliable quality were available, grouped by clinical improvement at month 3 and presence of adequate treatment. Mean ± SD. | | | | | | |
| --- | --- | --- | --- | --- | --- | --- |
|  | All patients with FEP (n = 16) | | | Patients with FEP with adequate treatment (n = 11) | | |
|  | clinical improvement  *n* = 5 | no clinical improvement  *n*= 11 | *p* values | clinical improvement  *n* = 5 | no clinical improvement  *n* = 6 | *p* values |
| Caudate Nucleus | 16.7 ± 14.9 | 9.3 ± 7.5 | 0.3439 | 16.7 ± 14.9 | 6.2 ± 5.6 | 0.2001 |
| Putamen | 25.6 ± 16.3 | 17.9 ± 10.8 | 0.374 | 25.6 ± 16.3 | 17.8 ± 13.5 | 0.4212 |
| Ventral Striatum | 28.5 ±21.4 | 19.4 ± 10.8 | 0.4105 | 28.5 ±21.4 | 22.2 ± 6.5 | 0.5573 |
| Globus Pallidus | 24 ± 17.4 | 13.4 ± 11.6 | 0.2599 | 24 ± 17.4 | 14.4 ± 16.1 | 0.3688 |
| SNVTA | 29.6 ± 15.5 | 31.5 ± 18.5 | 0.8363 | 29.6 ± 15.5 | 43.5 ± 10.2 | 0.131 |

| **Supplementary Table 2.** Correlation coefficients of baseline [^11^C]-(+)-PHNO BP_ND_ in different brain regions and PANSS score reduction | | | | | | |
| --- | --- | --- | --- | --- | --- | --- |
|  | | PANSS total score reduction | | | | |
|  | Baseline PANSS | week 4 | week 6 | month 3 | month 6 | month 12 |
| Caudate Nucleus | -0.06 | 0.20 | 0.003 | 0.15 | 0.11 | 0.01 |
| Putamen | -0.06 | 0.33 | 0.10 | 0.17 | 0.04 | -0.25 |
| Ventral Striatum | -0.02 | 0.32 | 0.05 | 0.06 | 0.15 | 0.01 |
| Globus Pallidus | -0.16 | 0.07 | 0.05 | 0.27 | -0.12 | -0.04 |
| SN/VTA | -0.38 | -0.09 | -0.14 | 0.03 | -0.51 | -0.3 |
| * *p* < 0.05  ** *p* < 0.01 | | | | | | |
|  | | PANSS positive score reduction | | | | |
|  | Baseline PANSS | week 4 | week 6 | month 3 | month 6 | month 12 |
| Caudate Nucleus | 0.23 | 0.36 | 0.28 | 0.39 | 0.30 | 0.27 |
| Putamen | 0.02 | 0.33 | 0.18 | 0.30 | 0.002 | -0.07 |
| Ventral Striatum | 0.02 | 0.27 | 0.30 | 0.27 | 0.14 | 0.12 |
| Globus Pallidus | 0.05 | 0.22 | 0.24 | **0.50*** | 0.37 | 0.39 |
| SN/VTA | -0.23 | 0.08 | -0.02 | 0.27 | -0.08 | -0.09 |
| * *p* < 0.05  ** *p* < 0.01 | | | | | | |
|  | | PANSS negative score reduction | | | | |
|  | Baseline PANSS | week 4 | week 6 | month 3 | month 6 | month 12 |
| Caudate Nucleus | -0.28 | 0.10 | -0.10 | 0.02 | 0.04 | -0.18 |
| Putamen | 0.07 | 0.42 | 0.19 | 0.29 | 0.22 | -0.27 |
| Ventral Striatum | 0.10 | 0.16 | 0.18 | 0.10 | 0.08 | -0.12 |
| Globus Pallidus | -0.16 | -0.12 | -0.09 | 0.02 | -0.42 | -0.36 |
| SN/VTA | -0.15 | -0.35 | -0.25 | -0.19 | **-0.69*** | -0.48 |
| * *p* < 0.05  ** *p* < 0.01 | | | | | | |
|  | | PANSS general score reduction | | | | |
|  | Baseline PANSS | week 4 | week 6 | month 3 | month 6 | month 12 |
| Caudate Nucleus | -0.08 | 0.11 | -0.06 | 0.08 | 0.03 | -0.01 |
| Putamen | -0.16 | 0.20 | 0.04 | 0.02 | -0.07 | -0.28 |
| Ventral Striatum | -0.10 | 0.35 | 0.08 | 0.06 | 0.15 | 0.04 |
| Globus Pallidus | -0.22 | 0.07 | 0.03 | 0.22 | -0.12 | -0.07 |
| SN/VTA | -0.41 | -0.04 | -0.11 | 0.02 | -0.47 | -0.31 |
| * *p* < 0.05  ** *p* < 0.01 | | | | | | |
| **Supplementary Table 3.** Correlation coefficients of post-amphetamine [^11^C]-(+)-PHNO BP_ND_ in different brain regions and PANSS score reduction | | | | | | |
|  | | PANSS total score reduction | | | | |
|  | Baseline PANSS | week 4 | week 6 | month 3 | month 6 | month 12 |
| Caudate Nucleus | 0.001 | 0.05 | -0.12 | 0.20 | 0.32 | 0.20 |
| Putamen | 0.06 | -0.33 | -0.31 | -0.24 | -0.52 | -0.62 |
| Ventral Striatum | 0.09 | -0.26 | -0.20 | -0.32 | -0.23 | -0.35 |
| Globus Pallidus | -0.15 | -0.47 | -0.35 | -0.08 | -0.53 | -0.35 |
| SN/VTA | -0.12 | -0.11 | -0.12 | -0.03 | -0.44 | -0.32 |
| * *p* < 0.05  ** *p* < 0.01 | | | | | | |
|  | | PANSS positive score reduction | | | | |
|  | Baseline PANSS | week 4 | week 6 | month 3 | month 6 | month 12 |
| Caudate Nucleus | 0.31 | 0.10 | 0.23 | 0.40 | 0.33 | 0.32 |
| Putamen | -0.06 | -0.42 | -0.22 | -0.01 | -0.40 | -0.38 |
| Ventral Striatum | -0.15 | **-0.55*** | -0.26 | -0.35 | -0.53 | -0.48 |
| Globus Pallidus | -0.03 | -0.33 | -0.14 | 0.24 | -0.03 | 0.01 |
| SN/VTA | -0.02 | 0.08 | -0.02 | 0.33 | 0.002 | -0.03 |
| * *p* < 0.05  ** *p* < 0.01 | | | | | | |
|  | | PANSS negative score reduction | | | | |
|  | Baseline PANSS | week 4 | week 6 | month 3 | month 6 | month 12 |
| Caudate Nucleus | -0.23 | 0.01 | -0.20 | 0.08 | 0.12 | -0.08 |
| Putamen | **0.5*** | 0.13 | -0.05 | -0.10 | -0.44 | -0.66 |
| Ventral Striatum | 0.4 | 0.02 | 0.10 | -0.09 | -0.02 | -0.15 |
| Globus Pallidus | 0.17 | **-0.52*** | -0.30 | -0.26 | **-0.77*** | -0.48 |
| SN/VTA | 0.15 | -0.09 | -0.01 | -0.09 | -0.46 | -0.36 |
| * *p* < 0.05  ** *p* < 0.01 | | | | | | |
|  | | PANSS general score reduction | | | | |
|  | Baseline PANSS | week 4 | week 6 | month 3 | month 6 | month 12 |
| Caudate Nucleus | -0.08 | 0.03 | -0.1 | 0.15 | 0.30 | 0.20 |
| Putamen | -0.14 | -0.28 | -0.25 | -0.26 | -0.44 | -0.56 |
| Ventral Striatum | 0.02 | -0.13 | -0.10 | -0.21 | -0.11 | -0.27 |
| Globus Pallidus | -0.33 | -0.41 | -0.34 | -0.10 | -0.46 | -0.40 |
| SN/VTA | -0.26 | -0.18 | -0.18 | -0.18 | -0.50 | -0.37 |
| * *p* < 0.05  ** *p* < 0.01 | | | | | | |

| **Supplementary Table 4.** Correlation coefficients of post-amphetamine [^11^C]-(+)-PHNO BP_ND_ in different brain regions and PANSS negative scores | | | | | | |
| --- | --- | --- | --- | --- | --- | --- |
|  | | PANSS negative score r | | | | |
|  | Baseline PANSS | week 4 | week 6 | month 3 | month 6 | month 12 |
| Caudate Nucleus | -0.12 | 0.10 | -0.22 | -0.13 | -0.34 | -0.20 |
| Putamen | **0.59*** | **0.60*** | 0.43 | 0.44 | **0.62*** | **0.79**** |
| Ventral Striatum | 0.38 | 0.40 | 0.45 | 0.42 | 0.28 | 0.42 |
| Globus Pallidus | 0.22 | 0.46 | 0.32 | 0.32 | **0.70*** | 0.35 |
| SN/VTA | 0.19 | 0.14 | 0.02 | 0.15 | 0.44 | 0.32 |
| * *p* < 0.05  ** *p* < 0.01 | | | | | | |

| **Supplementary Table 5.** Correlation coefficients of dopamine release in different brain regions and PANSS score reduction | | | | | | |
| --- | --- | --- | --- | --- | --- | --- |
|  | | PANSS total score reduction | | | | |
|  | Baseline PANSS | week 4 | week 6 | month 3 | month 6 | month 12 |
| Caudate Nucleus | -0.10 | 0.43 | 0.30 | 0.26 | 0.01 | -0.07 |
| Putamen | -0.19 | **0.56*** | 0.31 | 0.35 | 0.30 | 0.12 |
| Ventral Striatum | -0.18 | **0.53*** | 0.19 | 0.33 | 0.28 | 0.28 |
| Globus Pallidus | 0.05 | **0.78**** | **0.67**** | **0.63*** | 0.53 | 0.42 |
| SN/VTA | -0.26 | 0.36 | 0.27 | 0.37 | 0.23 | 0.25 |
| * *p* < 0.05  ** *p* < 0.01 | | | | | | |
|  | | PANSS positive score reduction | | | | |
|  | Baseline PANSS | week 4 | week 6 | month 3 | month 6 | month 12 |
| Caudate Nucleus | 0.04 | **0.64*** | 0.29 | 0.29 | 0.24 | 0.18 |
| Putamen | 0.03 | **0.61*** | 0.30 | 0.29 | 0.21 | 0.18 |
| Ventral Striatum | 0.08 | **0.75**** | 0.47 | **0.54*** | 0.53 | 0.47 |
| Globus Pallidus | 0.06 | **0.76**** | **0.52*** | 0.36 | 0.39 | 0.42 |
| SN/VTA | -0.23 | 0.30 | 0.18 | 0.01 | 0.09 | 0.10 |
| * *p* < 0.05  ** *p* < 0.01 | | | | | | |
|  | | PANSS negative score reduction | | | | |
|  | Baseline PANSS | week 4 | week 6 | month 3 | month 6 | month 12 |
| Caudate Nucleus | 0.4033 | 0.40 | 0.11 | 0.12 | 0.06 | -0.22 |
| Putamen | 0.1424 | **0.63*** | 0.21 | 0.41 | 0.46 | 0.05 |
| Ventral Striatum | 0.2504 | 0.17 | 0.11 | 0.22 | 0.10 | 0.03 |
| Globus Pallidus | 0.3138 | **0.64*** | 0.42 | **0.58*** | 0.49 | 0.20 |
| SN/VTA | 0.2560 | -0.09 | -0.05 | 0.19 | 0.004 | 0.08 |
| * *p* < 0.05  ** *p* < 0.01 | | | | | | |
|  | | PANSS general score reduction | | | | |
|  | Baseline PANSS | week 4 | week 6 | month 3 | month 6 | month 12 |
| Caudate Nucleus | 0.7844 | 0.23 | 0.16 | 0.12 | -0.14 | -0.12 |
| Putamen | 0.6385 | 0.37 | 0.21 | 0.18 | 0.15 | 0.09 |
| Ventral Striatum | 0.4927 | 0.42 | 0.12 | 0.21 | 0.16 | 0.22 |
| Globus Pallidus | 0.5096 | **0.66**** | **0.60*** | **0.53*** | 0.44 | 0.42 |
| SN/VTA | 0.6705 | 0.47 | 0.37 | 0.51 | 0.35 | 0.35 |
| * *p* < 0.05  ** *p* < 0.01 | | | | | | |

Marder-5 factor Analysis for PANSS

We have assessed the association between D-amphetamine-induced dopamine released as measured by the displacement of [11C]-(+)-PHNO on symptom reduction on five symptom domains as proposed by Marder (Marder et al. European Neurophsychopharmacology 1997). We correlated the D-amphetamine induced dopamine release with the change in symptom scores from before treatment initiation to the 3 months follow-up time point. Overall the five factor model yielded slightly stronger associations with D-amphetamine-induced dopamine release at baseline as compared to the canonical sub-scales. In particular, treatment-response of the disorganized thought factor was strongly associated with a hyper-dopaminergic state before treatment initiation across brain regions.


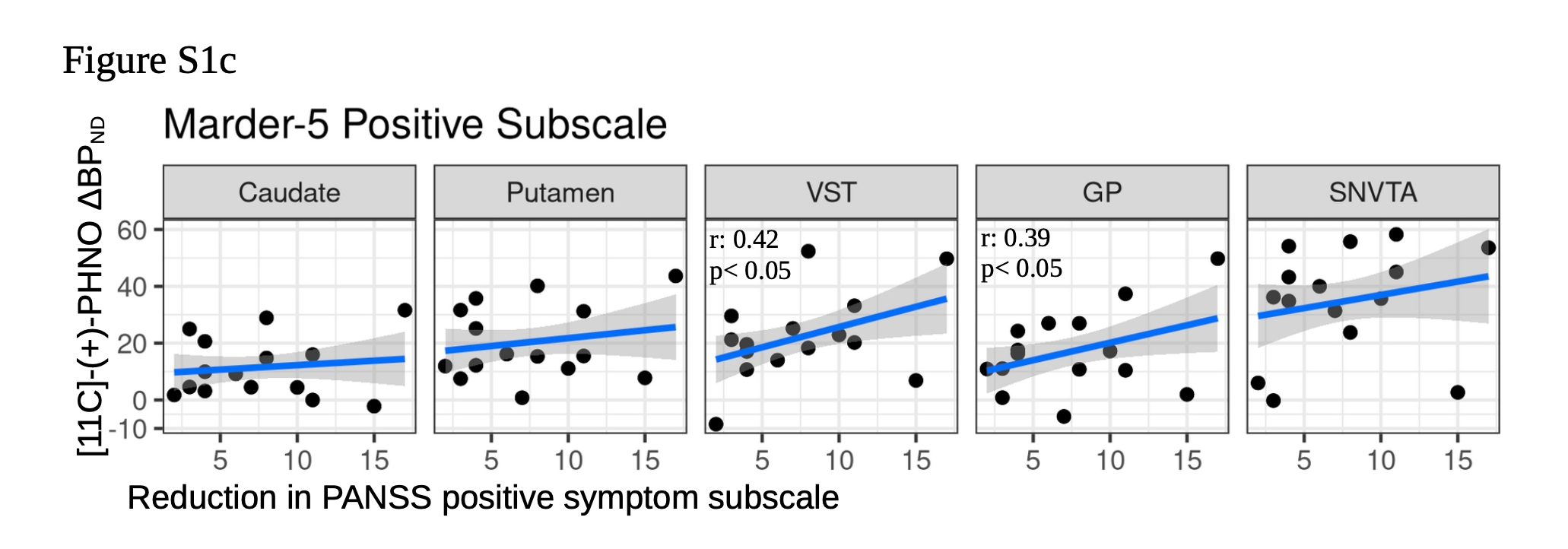


**Figure S1a.** Larger displacement of the radioligand [11C]-(+)-PHNO in the substantia nigra / ventral tegmental area (SN/VTA) (*p* = 0.00018, *r* = 0.55) due to D-amphetamine predicts larger reduction of the anxiety/depression factor of the PANSS.


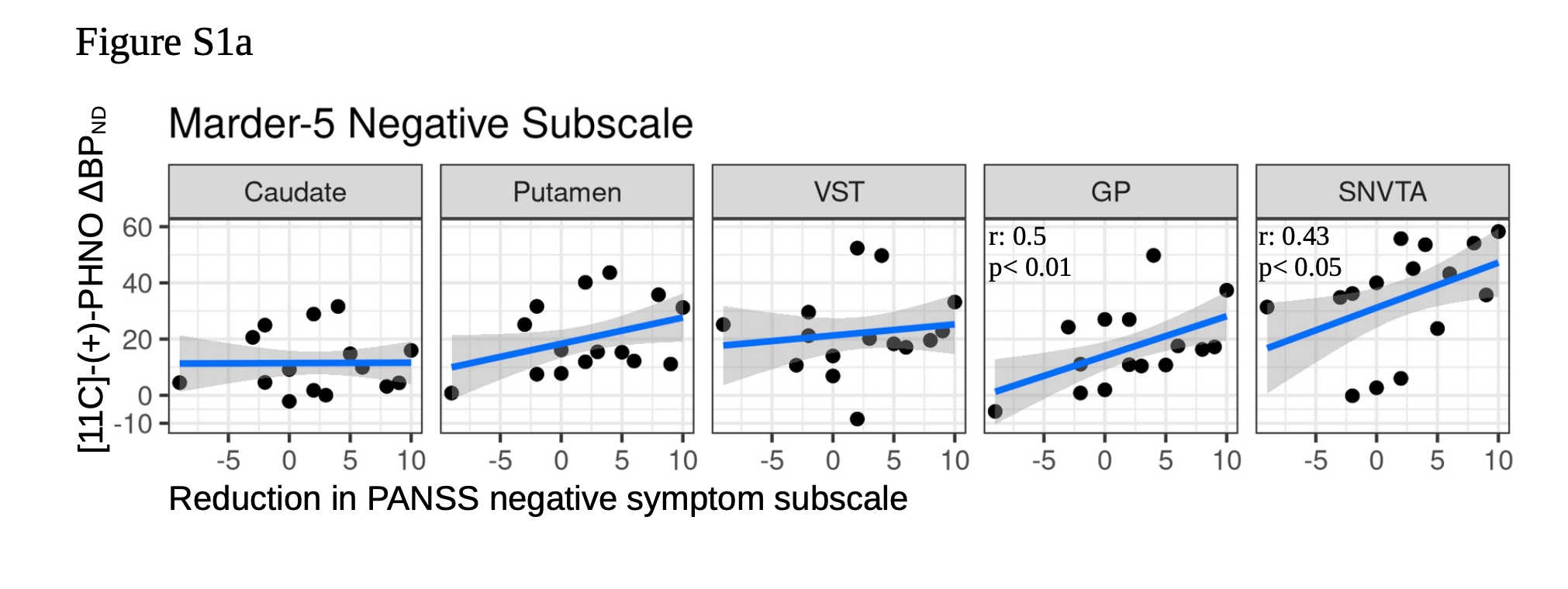


**Figure S1b.** Larger displacement of the radioligand [11C]-(+)-PHNO in the globus pallidus (*p* = 0.0048, *r* = 0.5) and the substantia nigra / ventral tegmental area (SN/VTA) (*p* = 0.019, *r* = 0.43) due to d- amphetamine predicts larger reduction of the negative symptoms factor of the PANSS.


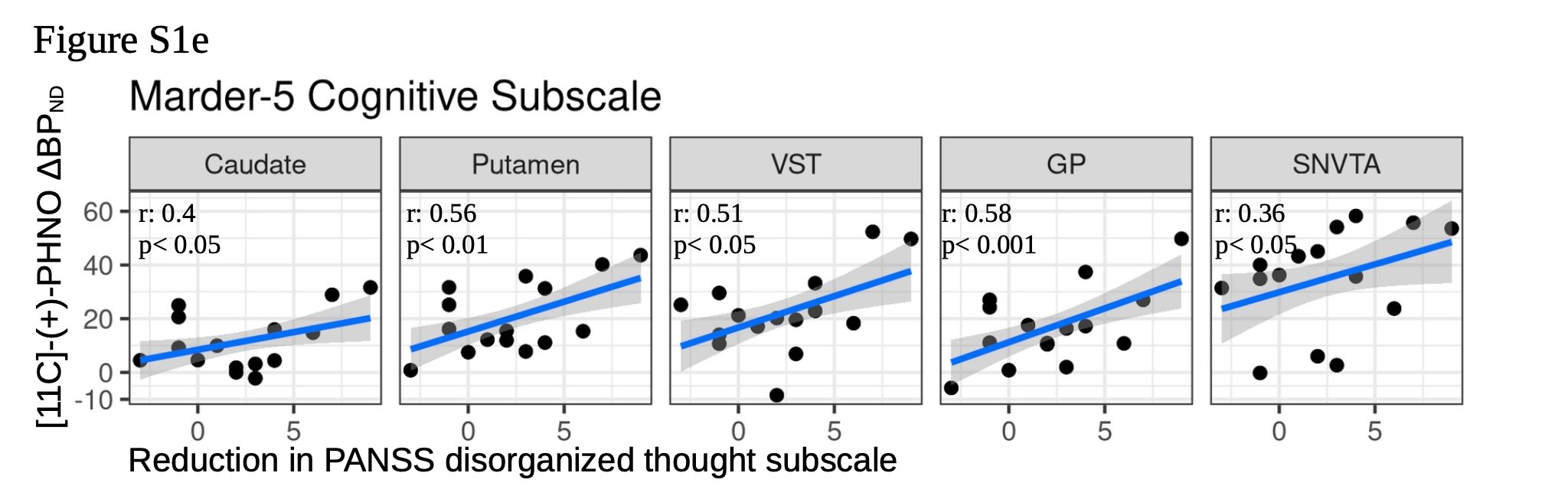


**Figure S1c.** Larger displacement of the radioligand [^11^C]-(+)-PHNO due to D-amphetamine predicts larger reduction of the disorganized thought factor of the PANSS in all subcortial dopaminergic brain areas investigated: caudate nucleus (*p* = 0.027, *r* = 0.4), putamen (*p* = 0.00013, *r* = 0.56), ventral striatum (VST) (*p* = 0.019, *r* = 0.51), globus pallidus (GP) (*p* = 0.0007, *r* = 0.58), and substantia nigra / ventral tegmental area (SNVTA) (*p* = 0.047, *r* = 0.036).


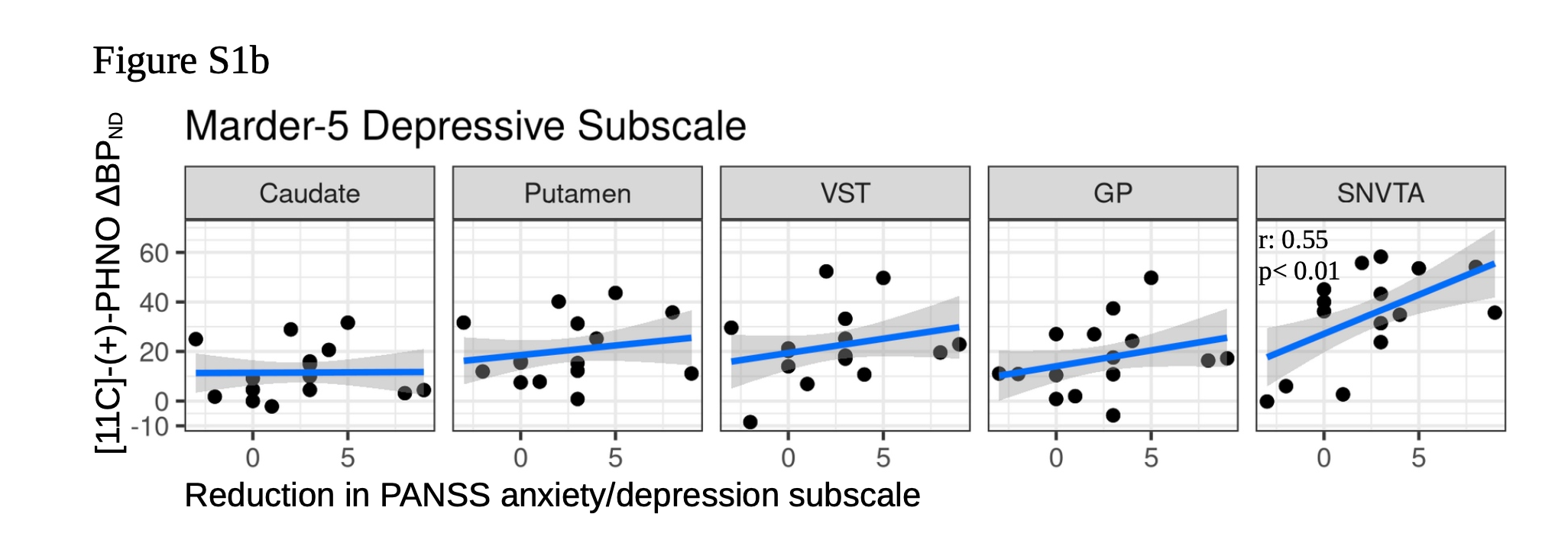


**Figure S1d**. Larger displacement of the radioligand [^11^C]-(+)-PHNO in the substantia nigra / ventral tegmental area (SNVTA) (*p* = 0.00018, *r* = 0.55) due to D-amphetamine predicts larger reduction of the anxiety/depression factor of the PANSS.


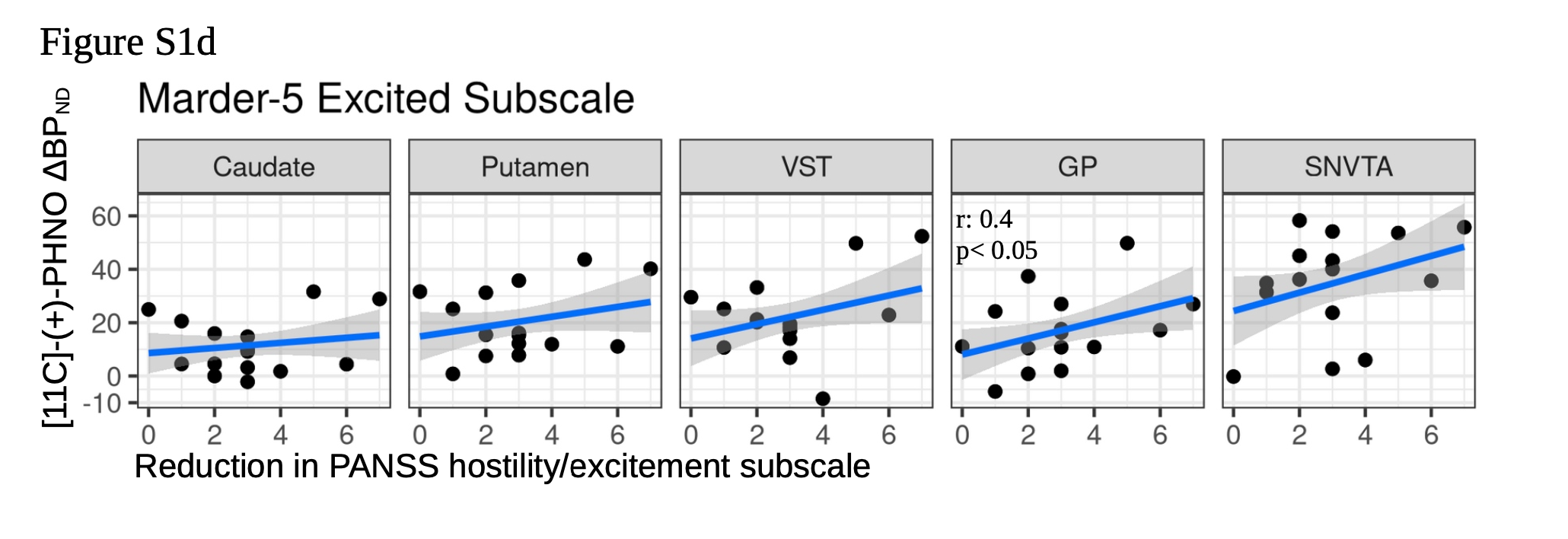


**Figure S1e**. Larger displacement of the radioligand [^11^C]-(+)-PHNO in the globus pallidus (GP) (*p* = 0.03, *r* = 0.4) due to d-amphetamine predicts larger reduction of the hostility/excitement factor of the PANSS as by the five factor solution of Marder et al. This association is found also in the ventral Striatum (VST) (*p* = 0.07, *r* = 0.33) and the Substantia nigra / ventral tegmental area (SNVTA) (*p* = 0.07, *r* = 0.34) where it does not reach the level of significance.


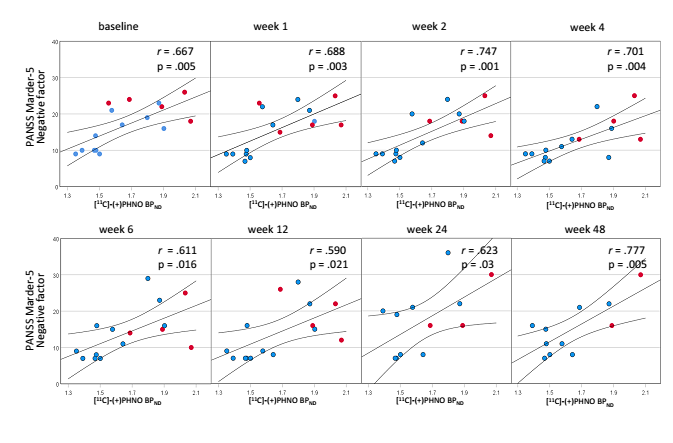


**Figure S2** depicts the correlation between [^11^C]-(+)-PHNO BP_ND_ values after D-amphetamine administration with the negative symptom factor from the Marder-5 factor solution of the PANSS obtained at baseline and during the follow up period and at the given time-points. The BP_ND_ value of [^11^C]-(+)-PHNO is inversely correlated with extracellular dopamine levels. These findings may be interpreted as larger expression of negative symptoms being associated with lower extracellular dopamine availability under hyperdopaminergic conditions due to D-amphetamine administration. Red dots indicate subjects with insufficient medication adherence.
